# On the torsion part in the *K*-theory of imaginary quadratic fields

**DOI:** 10.1007/s00229-024-01598-4

**Published:** 2024-10-09

**Authors:** Vincent Emery

**Affiliations:** Bern University of Applied Sciences, School of Engineering and Computer Science, Quellgasse 21, CH-2501 Bienne, Switzerland

**Keywords:** 14M25, 14C15, 14C17, 05E14

## Abstract

We obtain upper bounds for the torsion in the *K*-groups of the ring of integers of imaginary quadratic number fields, in terms of their discriminants.

## Introduction

Let *F* be a number field with ring of integers $\mathcal {O}_F$, and denote by $K_n(\mathcal {O}_F)$ Quillen’s *K*-theory group of degree *n*. By a theorem of Quillen $K_n(\mathcal {O}_F)$ is finitely generated, and its rank was computed by Borel for each $n \ge 2$ [4]. We refer to [13] for a detailed survey concerning the problem of computing these groups. In this paper we are interested in the problem of finding upper bounds for the torsion part of $K_n(\mathcal {O}_F)$. We obtain a result in the case of imaginary quadratic fields:

### Theorem 1.1

Let $n \ge 2$. There exists a constant *C*(*n*) such that for every imaginary quadratic number field *F*, the group $K_n(\mathcal {O}_F)$ contains no *p*-torsion element for any prime *p* with

1.1 $$\begin{aligned} \log (p) > C(n) |D_F|^{2n(n+1)}, \end{aligned}$$

where $D_F$ denotes the discriminant of *F*.

For $n = 2$ the better estimate

1.2 $$\begin{aligned} \log \left| K_2(\mathcal {O}_F) \otimes \mathbb {Z}\big [ 1/6 \big ] \right|&\le C \cdot |D_F|^2 \log |D_F| \end{aligned}$$

was obtained in [6]. Tensoring by $\mathbb {Z}[1/6]$ excludes the 2- and 3-torsion in the bound. In a similar way, Theorem 1.1 is obtained from an upper bound for $|\textrm{tors}\ K_n(\mathcal {O}_F)|$ that holds modulo small torsion, although we need to exclude more primes here (see Proposition 4.1).

Let us briefly indicate the strategy. It is classical that $K_n(\mathcal {O}_F)$ relates directly to the homology of ${{\,\textrm{GL}\,}}_N(\mathcal {O}_F)$, for $N = 2n+1$ (see Section 2). The general idea for the proof of Theorem 1.1 is to obtain an upper bound for the homology of ${{\,\textrm{GL}\,}}_N(\mathcal {O}_F)$ by using its action on the symmetric space $X = {{\,\textrm{PGL}\,}}_N(\mathbb {C})/{{\,\textrm{PU}\,}}(N)$. A theorem of Gelander (Theorem 3.2) controls the topology of noncompact arithmetic quotients of *X* in terms of their volume, and from this we can obtain an upper bound for the torsion homology of ${{\,\textrm{PGL}\,}}_N(\mathcal {O}_F)$ (Section 3). A spectral sequence argument finally provides the bound for $\textrm{tors}\, H_n({{\,\textrm{GL}\,}}_N(\mathcal {O}_F))$. Unfortunately the constant *C*(*n*) in (1.1) is not explicit, and its appearance is explained by some nonexplicit constant in Gelander’s theorem.

Our result can be compared with the upper bounds obtained by Soulé in [11, Prop.4 iii)]. He showed that for *F* imaginary quadratic one has “up to small torsion”:

1.3 $$\begin{aligned} \log |\textrm{tors}\, K_n(\mathcal {O}_F)|&\le |D_F|^{1120n^4 \log (n)}. \end{aligned}$$

Thus, Theorem 1.1 improves (asymptotically) the upper bound for the existence of *p*-torsion in $K_n(\mathcal {O}_F)$ that follows from (1.3). Soulé’s work also contains general upper bounds – without restriction on the signature of the number field *F* – which have been later improved in [2]. It is not clear if the strategy for Theorem 1.1 could be adapted to include the case of number fields with more than one archimedean places (see Remark 4.3). Until Section 4 and the last step in the proof, we will be treating the case of general number fields *F*.

A main motivation for the study of upper bounds in *K*-theory comes from the case $F = \mathbb {Q}$, for which the triviality of $K_{4m}(\mathbb {Z})$ is known to be equivalent to the Vandiver conjecture (cf. [10]). To our knowledge, the best available bounds (up to small torsion) for these groups are given in [11, Prop. 4 iv)]. The method for Theorem 1.1 applies directly to the case $F = \mathbb {Q}$, and an effective version of Gelander’s theorem would thus provide an upper bound for $K_n(\mathbb {Z})$. However, it might well be that the bounds obtained this way will be larger than Soulé’s bounds.

## Preliminaries

2.1 For an abelian group *A* we denote by $\textrm{tors}\, A$ its torsion subgroup. For $\ell > 1$, we define

2.1 $$\begin{aligned} \textrm{tors}_\ell \, A&= \textrm{tors}\left( A \otimes \mathbb {Z}\big [1/m\big ] \right) , \end{aligned}$$

where *m* is the lowest common multiple to all integers $\le \ell$. Thus, $\textrm{tors}_\ell$ kills all torsion up to $\ell$.

2.2 Let $\Gamma$ be a group. The symbol $H_n(\Gamma )$ will denote the homology of $\Gamma$ with coefficients in $\mathbb {Z}$. Since $\mathbb {Z}\left[ 1/m \right]$ is a flat $\mathbb {Z}$-module, it follows from the universal coefficient theorem [5, p. 8] that

2.2 $$\begin{aligned} \textrm{tors}_\ell \, H_n(\Gamma )&\cong H_n(\Gamma , \mathbb {Z}[1/m]), \end{aligned}$$

for *m* as above. In this context, we will also need the following fact.

### Lemma 2.1

Let $\Gamma _0 \subset \Gamma$ be a normal subgroup of index $[\Gamma : \Gamma _0] \le \ell < \infty$, and *m* be the lowest multiple common to all integers $\le \ell$. Then $H_n(\Gamma , \mathbb {Z}[1/m])$ injects into $H_n(\Gamma _0, \mathbb {Z}[1/m])$.

### Proof

Let $A = \Gamma / \Gamma _0$. Since $|A| = [\Gamma :\Gamma _0]$ is invertible in $\mathbb {Z}[1/m]$, the transfer map (cf. [5, §III.9–10]) gives an isomorphism between $H_n(\Gamma , \mathbb {Z}[1/m])$ and the module of co-invariants $H_n(\Gamma _0, \mathbb {Z}[1/m])_A$. But for |*A*| invertible, the norm map (cf. [5, §III.1]) provides an isomorphism of the latter with the submodule $H_n(\Gamma _0, \mathbb {Z}[1/m])^A$ of *A*-invariant elements in $H_n(\Gamma _0, \mathbb {Z}[1/m])$. $\square$

2.3 Let *F* denote a number field of degree *d*. Until Section 4 there is no restriction on *d*.

The group $K_n(\mathcal {O}_F)$ is defined as the *n*-th homotopy group $\pi _n(B{{\,\textrm{GL}\,}}(\mathcal {O}_F)^+)$ of Quillen’s plus contruction applied on the classifying space of the linear group ${{\,\textrm{GL}\,}}(\mathcal {O}_F) = \varinjlim {{\,\textrm{GL}\,}}_N(\mathcal {O}_F)$. Since the (integral) homology of $B {{\,\textrm{GL}\,}}(\mathcal {O}_F)^+$ is canonically isomorphic to the homology of ${{\,\textrm{GL}\,}}(\mathcal {O}_F)$, the Hurewicz map provides a homomorphism

2.3 $$\begin{aligned} K_n(\mathcal {O}_F)&\rightarrow H_n({{\,\textrm{GL}\,}}(\mathcal {O}_F)). \end{aligned}$$

The kernel of the Hurewicz map (2.3) does not contain *p*-torsion elements, for any $p > \frac{n+1}{2}$ (see [1]). It follows that for $\ell \ge \frac{n+1}{2}$, the group $\textrm{tors}_{\ell }\, K_n(\mathcal {O}_F)$ injects into $\textrm{tors}_\ell \, H_n({{\,\textrm{GL}\,}}(\mathcal {O}_F))$. Moreover, the stability result of Maazen (see van der Kallen [12, Theorem 4.11]) tells us that the homology group $H_n({{\,\textrm{GL}\,}}(\mathcal {O}_F))$ equals $H_n({{\,\textrm{GL}\,}}_N(\mathcal {O}_F))$ for any $N \ge 2n+1$. These various results provide the next proposition, which is already a basic ingredient in Soulé’s method [11].

### Proposition 2.2

For $N \ge 2n+1$ and $\ell \ge \frac{n+1}{2}$, there is an injective map

$$\begin{aligned} \textrm{tors}_{\ell }\, K_n(\mathcal {O}_F) \rightarrow \textrm{tors}_\ell \, H_n({{\,\textrm{GL}\,}}_N(\mathcal {O}_F)). \end{aligned}$$

## Torsion homology of ${{\,\textrm{PGL}\,}}_N(\mathcal {O}_F)$

3.1 Consider the semisimple Lie group $G = {{\,\textrm{PGL}\,}}_N(F \otimes _\mathbb {Q}\mathbb {R})$. The arithmetic subgroup $\Gamma = {{\,\textrm{PGL}\,}}_N(\mathcal {O}_F)$ is a nonuniform (irreducible) lattice in *G*. The following fact is well-known (see for instance [8, Lemma 13.1]):

### Lemma 3.1

There exist a constant $\gamma = \gamma (d,N)$ such that for any number field *F* of degree *d* the group $\Gamma = {{\,\textrm{PGL}\,}}_N(\mathcal {O}_F)$ has a torsion-free normal subgroup $\Gamma _0$ with index $[\Gamma : \Gamma _0 ] \le \gamma$.

The result essentially follows from Minkowski’s lemma that asserts that the reduction map ${{\,\textrm{GL}\,}}_n(\mathbb {Z}) \rightarrow {{\,\textrm{GL}\,}}(\mathbb {Z}/ m)$ has torsion-free kernel for $m > 2$. This permits to give an explicit value for the bound $\gamma$. See [3, Sect. 2] for an modified argument that provides better (i.e., lower) value for this constant.

3.2 Let *X* be the symmetric space associated with *G*, i.e, $X = G/K$ for some maximal compact subgroup $K \subset G$. Note that *X* depends only on *N* and the signature of *F*. For $\Gamma _0$ as in Lemma 3.1 the quotient $M = \Gamma _0 \backslash X$ is a noncompact locally symmetric space. Gelander’s theorem [8, Theorem 1.5 (1)] shows that *M* is homotopy equivalent to a simplicial complex of bounded size. Let us define a $(\delta , v)$-*simplicial complex* to be a simplicial complex with at most *v* vertices, all of valence at most $\delta$. Then the precise result is the following.

### Theorem 3.2

(Gelander)

There exist constants $\delta = \delta (X)$ and $\alpha = \alpha (X)$ such that any noncompact arithmetic manifold $M = \Gamma _0\backslash X$ is homotopically equivalent to a $(\delta , \alpha {{\,\textrm{vol}\,}}(M))$-simplicial complex $\mathcal {K}$.

### Remark 3.3

It is clear that the result holds for any normalization of the volume ${{\,\textrm{vol}\,}}$ on *X*, by adapting the constant $\alpha$ accordingly.

### Remark 3.4

The similar result for compact manifolds has been recently obtained in [7].

A result of Gabber allows to bound torsion homology of a simplicial complex. We will use this result in the following form.

### Proposition 3.5

Let $\mathcal {K}$ be a $(\delta , v)$-simplicial complex. Then the torsion homology of $\mathcal {K}$ is bounded as follows:

3.1 $$\begin{aligned} \nonumber \log |\textrm{tors}\, H_n(\mathcal {K})|&\le v \cdot \left( {\begin{array}{c}\delta \\ n\end{array}}\right) \\&\le v \cdot \delta ^n \end{aligned}$$

### Proof

The number of *n*-simplices in $\mathcal {K}$ is bounded above by $$\frac{v}{n+1} \left( {\begin{array}{c}\delta \\ n\end{array}}\right) $$. By Gabber’s lemma (cf. [6, Lemma 2.2]), since the chain complex $\mathcal {K}_\bullet$ is a *simplicial* complex we may bound the torsion homology as follows:

$$\begin{aligned} |\textrm{tors}\, H_n(\mathcal {K}_\bullet )|&\le \sqrt{n+2}^{\, \textrm{rank}(\mathcal {K}_n)}, \end{aligned}$$

from which the result follows. $\square$

3.3 From Theorem 3.2 and Proposition 3.5 we obtain

3.2 $$\begin{aligned} \log |\textrm{tors}\, H_n(\Gamma _0)|&\le \alpha \delta ^n \; {{\,\textrm{vol}\,}}(\Gamma _0 \backslash X) \nonumber \\&\le \alpha \delta ^n \gamma \; {{\,\textrm{vol}\,}}(\Gamma \backslash X), \end{aligned}$$

for constants $\alpha , \gamma$ and $\delta$ that can be chosen to depend only on *N* and *d*.

### Proposition 3.6

Let $N = 2n+1$ and $\Gamma = {{\,\textrm{PGL}\,}}_N(\mathcal {O}_F)$. For some constant *C*(*d*, *n*), we have the following upper bound for the torsion homology:

3.3 $$\begin{aligned} \log |\textrm{tors}_\gamma \, H_n(\Gamma )|&\le C(d,n) |D_F|^{2 n(n+1)}, \end{aligned}$$

where $\gamma = \gamma (d, 2n+1)$ is the constant in Lemma 3.1.

### Proof

By Section 2.2 and Lemma 2.1, it is enough to bound

$$\begin{aligned} |\textrm{tors}\,H_n(\Gamma _0, \mathbb {Z}[1/m])|&= |\textrm{tors}\, H_n(\Gamma _0) \otimes \mathbb {Z}[1/m]|\\&\le |\textrm{tors}\, H_n(\Gamma _0)|. \end{aligned}$$

Using (3.2), it remains to bound the covolume of $\Gamma = {{\,\textrm{PGL}\,}}_N(\mathcal {O}_F)$. It is certainly bounded by the covolume of ${{\,\textrm{PSL}\,}}_N(\mathcal {O}_F)$. We can choose (cf. Remark 3.3) to work with the normalization of the volume used in Prasad’s volume formula: let us assume that

3.4 $$\begin{aligned} {{\,\textrm{vol}\,}}({{\,\textrm{PSL}\,}}_N(\mathcal {O}_F)\backslash X) = \mu ({{\,\textrm{SL}\,}}_N(\mathcal {O}_F) \backslash \tilde{G}), \end{aligned}$$

where $\mu = \mu _\infty = \mu _S$ is the Haar measure on $\tilde{G} = {{\,\textrm{SL}\,}}_N(F \otimes _\mathbb {Q}\mathbb {R})$ defined in [9, §3.6]. Then the volume formula [9, Theorem 3.7] shows that

$$\begin{aligned} {{\,\textrm{vol}\,}}({{\,\textrm{PSL}\,}}_N(\mathcal {O}_F) \backslash X)&= A \cdot |D_F|^{\frac{N^2-1}{2}} \prod _{k=2}^{N} \zeta _F(k), \end{aligned}$$

for some (explicit) constant *A* that depends only on *N* and the signature of *F*. The product of zeta functions is easily shown to be $\le 2^{N-1}$, so that the result follows. $\square$

### Remark 3.7

Note that if we had explicit constants $\alpha$ and $\delta$ in Theorem 3.2 (for some explicit normalization of the volume), the procedure would permit to make the constant *C*(*d*, *n*) explicit.

## Upper bound in the *K*-groups

We finally state and prove the following result, of which Theorem 1.1 will be an immediate consequence (see proof below). Recall that the notation $\textrm{tors}_\gamma$ was introduced in Section 2.1.

### Proposition 4.1

Let $n \ge 2$. There are constants *C*(*n*) and $\gamma = \gamma (n)$ such that for any imaginary quadratic field *F*, one has

4.1 $$\begin{aligned} \log |\textrm{tors}_\gamma \, K_n(\mathcal {O}_F)|&\le C(n) |D_F|^{2n(n+1)}. \end{aligned}$$

### Proof

With $\gamma$ as in Lemma 3.1 and larger than $\frac{n+1}{2}$, we have from Proposition 2.2:

4.2 $$\begin{aligned} |\textrm{tors}_\gamma \, K_n(\mathcal {O}_F)|&\le |\textrm{tors}_\gamma \, H_n({{\,\textrm{GL}\,}}_N(\mathcal {O}_F))| \nonumber \\&= |H_n({{\,\textrm{GL}\,}}_N(\mathcal {O}_F), \mathbb {Z}[1/m])|, \end{aligned}$$

with $N = 2n+1$ and *m* the lowest multiple common to all integers $\le \gamma$.

With $\Gamma = {{\,\textrm{PGL}\,}}_N(\mathcal {O}_F)$, we have the short exact sequence

4.3 $$\begin{aligned} 1&\rightarrow \mathcal {O}_F^\times \rightarrow {{\,\textrm{GL}\,}}_N(\mathcal {O}_F) \rightarrow \Gamma \rightarrow 1, \end{aligned}$$

and for it the Lyndon-Hochschild-Serre spectral sequence (cf. [5, §VII.6]) reads:

4.4 $$\begin{aligned} E_{pq}^2 = H_p\left( \Gamma , H_q(\mathcal {O}_F^\times , \mathbb {Z}[1/m]) \right)&\implies H_{p+q}\left( {{\,\textrm{GL}\,}}_N(\mathcal {O}_F), \mathbb {Z}[1/m] \right) . \end{aligned}$$

Since we assume that *F* is imaginary quadratic, we have that $\mathcal {O}_F^\times$ is finite, of order $\le 6$. Then $H_q(\mathcal {O}_F^\times , \mathbb {Z}[1/m]) = 0$ for $q > 0$, and the spectral sequence is concentrated in $q = 0$. Note also that since the image of $\mathcal {O}_F^\times$ is central in ${{\,\textrm{GL}\,}}_N(\mathcal {O}_F)$, the action of $\Gamma$ on $H_0(\mathcal {O}_F^\times , \mathbb {Z}[1/m]) = \mathbb {Z}[1/m]$ is trivial (cf. [5, ex. 1 p.80]). We obtain:

$$\begin{aligned} H_n(\Gamma , \mathbb {Z}[1/m])&= H_n({{\,\textrm{GL}\,}}_N(\mathcal {O}_F), \mathbb {Z}[1/m]), \end{aligned}$$

and the result follows from Proposition 3.6 with the same constant $C(n) = C(2, n)$. $\square$

### Remark 4.2

As it follows from the discussion in Section 3.1, the constant $\gamma$ can be easily made explicit – contrarily to *C*(*n*).

### Remark 4.3

For *F* with more than one archimedean place, the spectral sequence (4.4) does not collapse at $E^2_{pq}$. A priori the differential $\partial _r$ (with $r \ge 2$) might add torsion, whenever both its domain and image is infinite. It seems difficult to control this phenomena to obtain upper bounds in this more general setting.

### Proof of Theorem 1.1

In Proposition 4.1, we can assume that $C(n) \ge \log (\gamma )$ (by increasing the constant if necessary). Let $s \in K_n(\mathcal {O}_F)$ be an element of oder *p*, with *p* prime such that $\log (p) > C(n) |D_F|^{2n(n+1)}$. In particular, we have $p > \gamma$, so that the image of *s* in $\textrm{tors}_\gamma \, K_n(\mathcal {O}_F)$ is non-trivial. But this contradicts the bound (4.1).

## Declarations

### Data availability

This work is purely based on a theoretical approach; no datasets were used.
